# One-step extraction and analysis of 45 contaminants of emerging concern using QuEChERS methodology and HR-MS in radish leaves and roots

**DOI:** 10.1016/j.mex.2021.101308

**Published:** 2021-03-15

**Authors:** F. Labad, N. Montemurro, S. Berisha, N.S. Thomaidis, S. Pérez

**Affiliations:** a ENFOCHEM^,^ Deptartment of Environmental Chemistry, IDAEA-CSIC, Jordi Girona 18-26 08034, Barcelona, Spain; b Deptartment of Chemistry, National and Kapodistrian University of Athens, Zografou 157 84, Greece

**Keywords:** QToF-MS, CECs, Radish, MRMHR

## Abstract

The scarcity of freshwater has led to a considerable increase of the reuse of reclaimed wastewater for irrigation of field crops [1,2]. This practice potentially exposes agricultural produce to a large variety of xenobiotic compounds including contaminants of emerging concern (CECs) which have been widely recognized to be present in wastewater [3]. Common approaches for the extraction of CECs from crops rely on solid-liquid extraction [4], assisted solvent extraction [5], ultra-sound solvent extraction [6] and recently QuEChERS (QUick, Easy, CHeap, Effective, Rugged and Safe) [[7], [8]–9]. Here, eight QuEChERS-based methodologies were compared for their suitability to determine 45 CECs in roots and leaves of soil-grown radish.

The key points of the method development were:

• The development of two single-step analytical methods specific for radish root and leaves, after testing eight different approaches using QuEChERS extraction for the quantitation of 45 CECs. The analytical methodology selected requires minimal time and solvent, making it cost-effective.
• Methods validation were performed at five concentrations levels (2, 5, 10, 50 and 200 ng g^−1^), with low limits of quantification between 0.01 and 0.32 ng g^−1^.
• The two optimized methodologies may be applied to identify large number of compounds of different families in radish crop. However, validation will be needed to quantify compounds different from the target compounds of this paper.

## Graphical abstract

**Figure:**
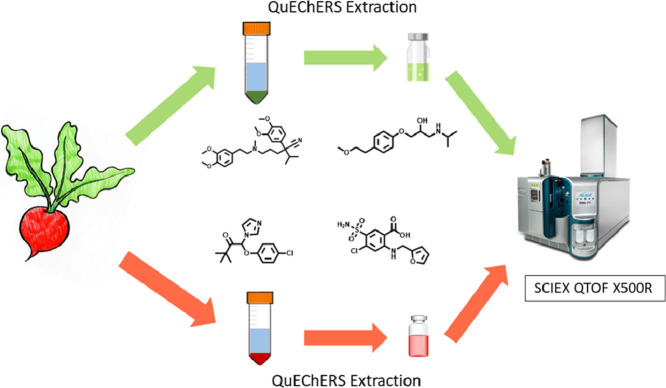

Specifications table

**Table:** 

Subject Area:	Chemistry
More specific subject area:	Environmental Analytical Chemistry
Method name:	Combination of one-step extraction method and HRMS used for the quantitation of 45 CECs in radish root and leaves.
Name and reference of original method:	Comparison of high resolution MRM and Sequential Window Acquisition of all Theoretical fragment-ion acquisition modes for the quantitation of 48 wastewater-borne pollutants in lettuce [7].
Resource availability:	*SCIEX O.S. V.1.6 or higher*

## Method details

The extraction of contaminants of emerging concern (CECs) from plants tissues is a challenging procedure due to the complexity of the matrix. Plants, including crops, contain lipids, proteins, fatty acids and a wide range of components [10,11] that could negatively interfere with the analytical performance, particularly in the ionization of MS-based detection [12]. In lasts years, the QuEChERS use for the extraction of organic compounds in biological matrices has grown. Although originally developed for pesticide residues analysis in food stuff [13], some method modifications have been implemented in order to adapt QuEChERS to extract diverse compounds of interest from various matrices [14]. Here, after testing several QuEChERS variants, two simple but efficient approaches were developed to extract CECs from root and leaves of radish. The differences between roots and leaves matrices called for the use of two QuEChERS methods differing in the salt composition: for roots, the original extraction salts (OR), also known as non-buffered salts while CEN 15662 (EN) the buffered-salts was chosen for leaves. Our validated analytical methodology is time-efficient, due to the reduced number of steps in the sample treatment, uses little organic solvent, and is sensitive enough to detect trace levels in radish by using LC-QToF-MS. Target analytes were selected among the most reported CECs in reclaimed wastewater and taking into account their wide diversities in terms of physico-chemical properties.

## Matrix preparation

For validation purposes, bunches of radish plants (leaves and roots) were bought from a local organic supermarket (Barcelona, Spain). Then, the whole plants were carefully hand-washed to remove any soil particles and roots were separated from leaves and frozen for at -20  °C for 48h, separately. Consequently, the tissues were lyophilized (LyoAlfa 6 system, Telstar Technologies, Terrassa, Spain) and ground to powder using a knife mill (Grindomix GM 200, Retsch, GmbH, Haan, Germany) and finally stored at -20  °C for the method development.

## Materials and reagents

Reference standards (purity > 90%) of the 45 target compounds: acesulfame, acetaminophen, acridone, benzotriazole, bezafibrate, bisphenol A, caffeine, carbamazepine, chloramphenicol, ciprofloxacin, citalopram, clarithromycin, climbazole, clofibric acid, diclofenac, diltiazem, fenofibrate, fluconazole, furosemide, gemfibrozil, hydrochlorothiazide, ibuprofen, irbesartan, indomethacin, lamotrigine, metoprolol, metronidazole, propranolol, sucralose, sulfamethazine, sulfamethoxazole, sulfanilamide, sulfanilic acid, valsartan, verapamil, 4-nitro-sulfamethoxazole, 4-hydroxy-diclofenac, 5-des-5-oxo-lamotrigine, 5-methyl-benzotriazole, carbamazepine epoxide, lamotrigine-N2-oxide, N-acetyl-sulfamethoxazole, N2-methyl-lamotrigine, oxcarbazepine and valsartan acid, were purchased from Sigma Aldrich (St. Louis, MO, US). Isotope-labelled compounds used as surrogates (IS), were purchased from Toronto Research Chemicals (Toronto, ON, Canada), Sigma Aldrich (St. Louis, MO, US), Santa Cruz Biotechnology (Dallas, TX, US) and Alsachim (Illkirch-Graffenstaden, France): acetaminophen-d_4_, acesulfame-d_4_, bisphenol A-d_8_, citalopram-d_8_, diclofenac-^13^C_6_, fenofibrate-d_6_, gemfibrozil-d_6_, hydrochlorothiazide-d_6_, furosemide-d_5_, benzotriazole-d_4_, bezafibrate-d_4_, indomethacin-d_4_, lamotrigine-^13^C_3_, sucralose-d_6_, fluconazole-^13^C_3_, carbamazepine-d_10_, climbazole-d_4_, irbesartan-d_6_, caffeine-^13^C_3,_ ciprofloxacin-d_8_, metoprolol-d_7,_ metronidazole-d_4_, sulfamethoxazole-d_4_, sulfamethazine-d_4_, and valsartan-d_3_, valsartan acid-d_4_, Stock solutions of the standards were prepared in methanol at 2  mg mL^−1^ and the cocktail of IS was prepared by dilution starting from a mix of 2  µg mL^−1^ in methanol and were stored at -20  °C. LC-MS grade acetonitrile (ACN) (≥ 99.9%), methanol (MeOH) (≥99.9%), and HPLC water were acquired from Merck (Darmstadt, Germany). Formic acid (HCOOH) (≥ 96%, ACS reagent), acetone (≥ 96%, ACS reagent) and ammonium acetate were supplied by Sigma Aldrich (St. Luis, MO, U.S). For the preparation of the mobile phases, ammonium fluoride; ACN and water (Optima™ LCMS Grade) were obtained from Fisher Chemical (Fisher Scientific SL, Madrid, Spain). Their extraction salts employed, Original non-buffered QuEChERS salt (OR), buffered CEN 15662 QuEChERS salt (EN) as well as dispersive solid-phase extraction (dSPE) clean-up mixture, were purchased from Bekolut GmbH & Co. KG (Hauptshul, Germany). CAS numbers, molecular formulas, molecular weight, and relevant physico-chemical properties of all target compounds are compiled in the previous studies [15,16].

## Extraction procedure

**Fig. 1:**
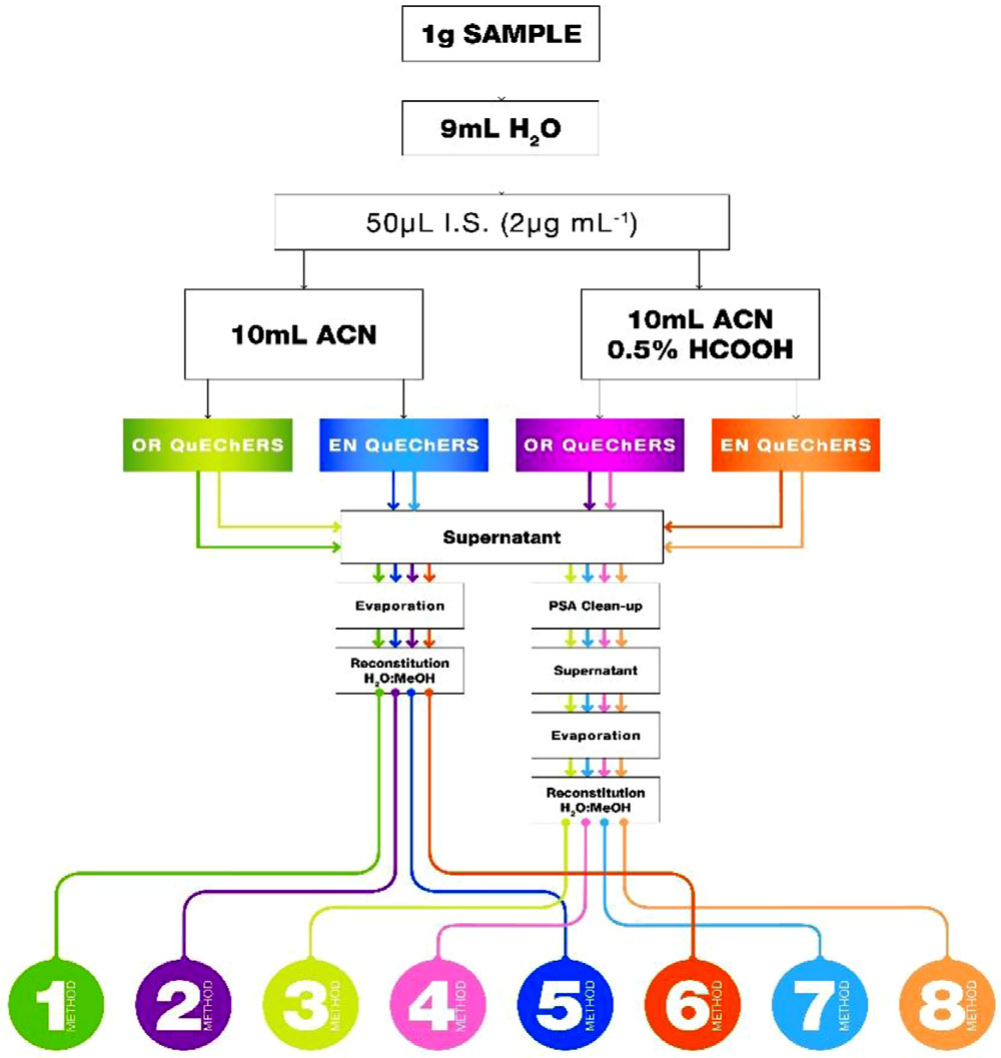
Schematic step-by-step procedures for the eight QuEChERS methodologies studied.

Eight QuEChERS analytical protocols were tested with two different extraction solvents with and without addition of 0.5% HCOOH, two QuEChERS salts and one dSPE clean-up (Fig. 1) They were applied to roots and leaves selecting the best approach for each matrix. All tests were performed in triplicate (n=3). The original method by Anastassiades et.al., was modified as follows: First, 1g of previously lyophilized and ground samples, were placed into a 50 mL polypropylene centrifuge tube. The sample was hydrated adding 9  mL of water and vortexed for 2  min. After 1  h, 50  µL standard mixture (2  µg mL^−1^ in MeOH) were added to achieve a final concentration of 10  ng g^−1^. Then, sample was vortexed for 5  min and then allowed to stand for 1 h. Next, 10  mL of extraction solvent were added and the sample was vortexed for 2  min. QuEChERS salt was added, and the tube was hand-shaken in order to avoid the formation of MgSO_4_ agglomerates followed by another vortex. The sample was centrifuged for 10  min at 4000  rpm at 4  ˚C, the organic phase (top layer) was transferred to a glass tube and kept overnight at -20  °C to induce the precipitation fatty acids, proteins, chlorophyll and sugars, which would have interfered on the analysis [17,18]. Six milliliters of the organic phase were carefully aspirated and transferred directly into the PSA-containing tube (900 mg MgSO_4_, 150 mg PSA, 150 mg C18), immediately hand-shaken for 30  s and vortexed for 2  min. Next, the suspension was centrifuged for 5  min at 4000 rpm at 4 °C. Finally, 1 mL supernatant was transferred into a HPLC glass vial. Then, sample was evaporated until dryness under a gentle N_2_ stream and reconstituted in 1  mL of 10% MeOH prior to LC-MS/MS analysis. In method 1, 3, 5 and 7, after keeping extracts at -20  °C overnight, 1 mL of the organic layer (supernatant) were analyzed without the clean-up step. In the selected methods, method 3 and 5, no clean-up step was considered as greater recoveries were achieved.

## LC-MS/MS acquisition and data analysis

**Fig. 2:**
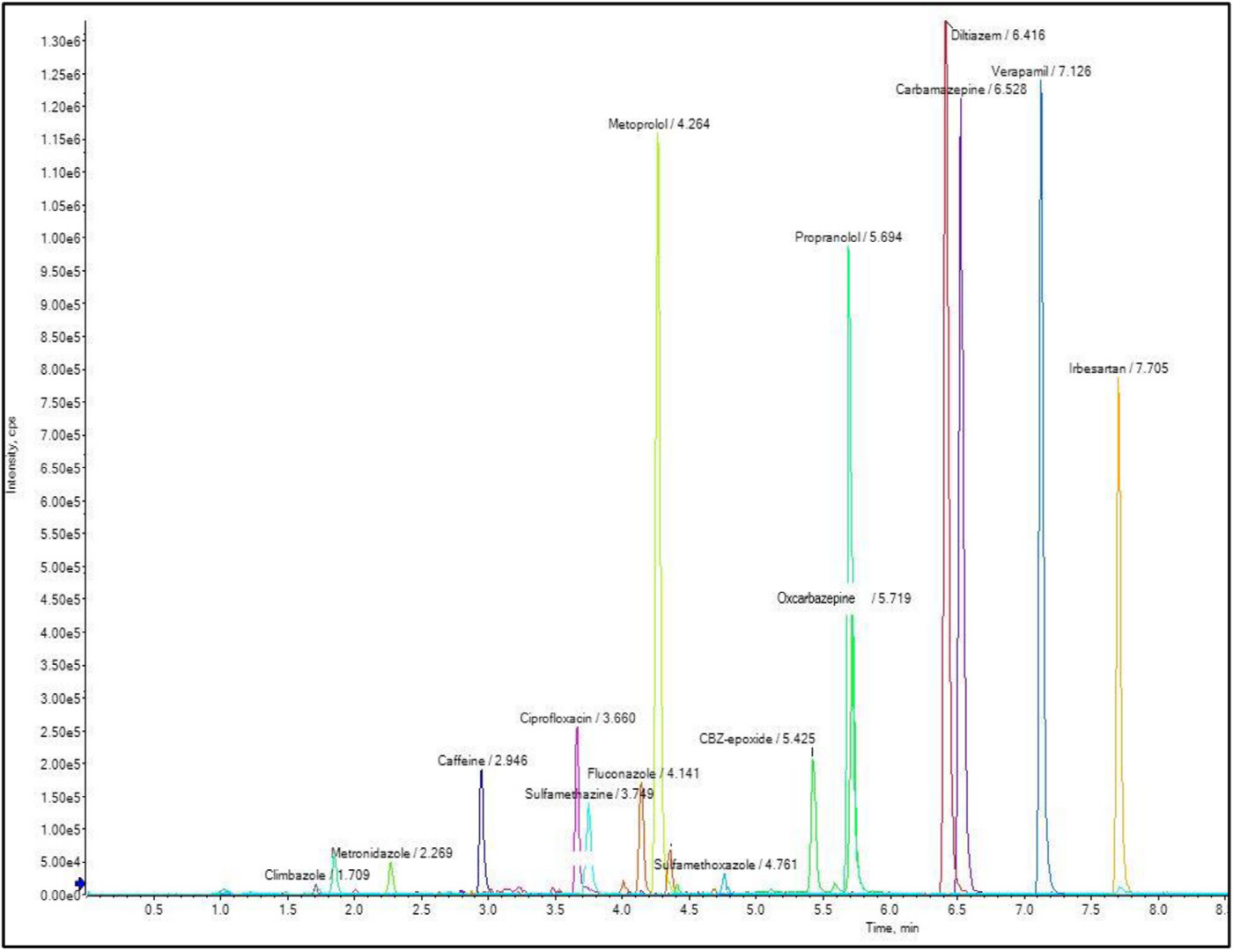
Example of chromatogram for validated CECs in positive ESI polarity, spiked at 50 ng g-1 USAR MUESTRA REAL O BORRARUSAR.

Analysis of final extracts was performed on SCIEX ExionLC™ AD chromatograph coupled with a SCIEX X500R QTOF (Sciex, Redwood city, CA, U.S.) with Turbo V™ electrospray Ionization (ESI). The ion mode was selected based on highest sensitivity of the molecular ion. The injection volume was 10  µL with an auto-sampler temperature set to 8  °C. The chromatographic run time was 13  min and chromatographic separation was achieved on a Hibar^Ⓡ^ HR Purospher^Ⓡ^ STAR RP-C18 column (100 mm x 2.1  mm i.d., 2  µm particle size, Merck, Darmstadt, Germany), at 40  °C. Mobile phases +ESI and -ESI were (A) 5  mM ammonium acetate with 0.1% formic acid and (B) ACN and (A) 2  mM ammonium fluoride in water and (B) ACN, respectively. The flow rate was 0.5  mL/min. The elution gradient was programmed as follows: with 5 % B (0.0 min)-5 % (0.33  min)-40 % (6.33  min)-96 % (10.33  min)-96 % (11.16  min) and 5 % (13.16  min). Chromatographic separation of target compounds in positive ionization is showed on Fig. 2.

The settings of the ion source were as follows: ion spray voltage was set to 5500 and -4500 V for (+ESI) and (-ESI), respectively; source temperature and nitrogen gas flows were set to 550°C and 55 psi, respectively, curtain gas 30 psi, and collision gas (CAD) 7. For MRM^HR^ the precursor ion selection consisted of one TOF-MS survey (100-950 Da for 120 ms of Accumulation time (AT); Declustering Potential (DP) and Collision Energy (CE) were set to 80 and 10 V and -80 and -10 V, for (+)ESI and (-)ESI, respectively. The Guided MRM^HR^ tool from SCIEX was used for optimizing the high-resolution transitions (see Table 1).

**Table 1:** LC-ESI-MSMS optimized detection parameters for target analytes for radish root and radish leaves.

	Analyte	RT (min)	Experimental Precursor ion (m/z)	Experimental Fragment ion (m/z)
(+)ESI				
2	5-Desamino 5-oxo-2,5-dihydro lamotrigine	4.65	256.9991	228.9985
3	5-Methyl-2H-benzotriazole	4.42	134.0713	77.0344
4	Acridone	5.85	196.0757	167.0644
5	Caffeine	2.83	195.0877	138.0685
6	Carbamazepine	6.44	237.1022	194.0981
7	Carbamazepine-10,11-epoxide	5.33	253.0972	180.0736
8	Ciprofloxacin	3.55	332.1405	314.1292
9	Citalopram	6.18	325.1711	109.0396
10	Clarithromycin	7.19	748.4842	158.1160
11	Diltiazem	6.33	415.1686	178.0261
12	Fenofibrate	9.89	361.1201	139.0002
13	Fluconazole	4.03	307.1113	238.0834
14	Lamotrigine	4.00	256.0151	210.9719
15	Lamotrigine N2-oxide	3.92	272.0106	242.0051
16	Metoprolol	4.16	268.1907	116.1077
17	Metronidazole	2.18	172.0717	128.0429
18	N2-Methyl-lamotrigine	4.59	270.0308	57.0390
1	4-Nitro-sulfamethoxazole	4.30	284.0336	189.0202
19	Oxcarbazepine	5.62	253.0972	180.0726
20	Propranolol	5.60	260.1645	116.1090
21	Sulfamethazine	3.64	279.0910	124.0937
22	Valsartan acid	5.40	267.0877	206.0519
23	Verapamil	7.06	455.2904	165.0883

(-)ESI				

25	Acesulfame	1.31	161.9866	82.0297
26	Acetaminophen	2.01	150.0560	107.0379
27	Benzotriazole	3.22	118.0411	50.0037
28	Bezafibrate	5.34	360.1008	274.0648
29	Bisphenol A	7.39	227.1077	212.0845
30	Chloramphenicol	5.16	321.0050	152.0352
31	Climbazole	8.38	291.0906	67.0304
32	Clofibric acid	4.10	213.0324	126.9961
33	Diclofenac	6.91	294.0094	250.0205
34	Furosemide	4.39	329.0004	285.0115
35	Gemfibrozil	8.76	249.1496	121.0671
36	Hydrochlorothiazide	2.77	295.9572	268.9476
24	4′-Hydroxydiclofenac	5.54	310.0043	266.0143
37	Ibuprofen	7.64	205.1234	161.1341
38	Indomethacin	7.20	356.0695	312.0798
39	Irbesartan	6.45	427.2247	193.1347
40	N-Acetylsulfamethoxazole	3.91	294.0554	198.0233
41	Sucralose	3.33	395.0073	359.0283
42	Sulfamethoxazole	4.25	252.0448	156.0131
43	Sulfanylamide	1.28	172.0069	79.9591
44	Sulfanilic acid	0.49	171.0229	78.9205
45	Valsartan	7.90	434.2197	179.0863

To maintain the mass accuracy of the MS detection, the instrument was automatically recalibrated during batch acquisitions by infusing reserpine reference standard (C_33_H_40_N_2_O_9_, m/z 609.2807) in (+ESI) or a cluster of sodium trifluoroacetate (detection of the cluster (TFA-Na)_5_^+^TFA^−^ at m/z 792.8596) for (-ESI). The instrument provided a resolving power at Full Width Half Maximum of between 31,000 and 44,000 at m/z 132.9049 and 829.5395 with a mass error of 0.4 ppm.

## Method validation

The protocols selected for validation were method 3 for roots and method 5 for leaves. It included the determination of accuracy, intra-day precision linearity, matrix effect (ME) and limits of detection (LOD) and quantification (LOQ). Accuracy was assessed in recovery studies for each compound at five spiking levels (n = 3) (2, 5, 10, 50 and 200 ng g^−1^). Recoveries were calculated as the ratio between the peak area in the extract from spiked radish root (or leaves) and the peak area in a blank radish root (or leaves) extract spiked at the same concentration levels. Blanks consisting of the radish tissues used in the validation study fortified only with the IS mixture were also analyzed to confirm the absence of the target analytes in this matrix. Intra-day precision or repeatability, was evaluated based on the relative standard deviation (RSD %) from the recovery data (n = 5). Good precision values were considered less than 20 % deviation for most of the compounds. Otherwise, LOD (the lowest concentration that could be distinguished of the matrix signal with a S/N greater than 3) and LOQ (the lowest concentration with a signal response that could be quantified with a S/N greater than 10 and an RSD ≤ 20%) were determined using the matrix matched calibration curves by linear regression [19,20]. To determine ME, blank matrixes (n = 3) were extracted following the selected protocol for both matrices and then spiked at the same concentration levels from the recovery studies. ME was determined comparing the peak areas from the spiked sample with peak areas from the standards in solvent (H_2_O/MeOH, 90:10, v/v) at the same concentration levels [21]. High influence of the matrix compounds in the analysis of an analyte was considered when ME was higher than ǀ40%ǀ [22]. Radish root and leaves are complex matrices due to the high effect of its compounds, resulting in high marked values in the ME. This phenomenon has been studied largely and it is well known that working with ESI the reduction of the signal is related with the ionization of the sample when it is transformed from liquid to gas and depending on the polarity of each compound the effect is different in every case [12,^23^,24]. The use of isotopically labelled Internal Standards (I.S.) serves for compensate the matrix effect (signal enhancement/suppression) but also improves accuracy and precision [25]. A matrix-matched calibration curve (CC) was elaborated using blank radishes and spiking them using at least eight different curve points, ranging from 0.05 to 300 ng mL^−1^ in dry weight radish. Linearity was accepted when coefficient correlation (r^2^) was ≥ 0.99. Table 2 shows the values obtained in the middle validation point (10 ng g^−1^), whereas Tables S1 and S2 contain the results of the other validation points. Globally, analytes were recovered satisfactory with a range between 70 and 120 % in most of the cases. However, overall recoveries of 5-methyl-2H-benzotriazole and carbamazepine-10,11-epoxide (53–94 and 25–82 %, respectively) were fairly low in roots at the 5 validation points. Otherwise, in leaves ciprofloxacin was the compound showing the lowest recoveries (13,14 %). The values of the ME differ between compounds but the CECs showing high enhancement in root were acetaminophen and 4-hydroxydiclofenac (421 and 451 %, respectively) whereas biggest signal suppression were observed in fenofibrate and gemfibrozil ((-93) and (-83), respectively). On the other hand, the highest signal enhancement in radish leaves were found in diltiazem (246 %) and oxcarbazepine (260 %) while great signal suppression were demonstrated in fenofibrate (99 %) and valsartan (94 %).

**Table 2:** Method validation parameters for radish roots and leaves spiked at 10 ng g^−1^ (MRMHR acquisition mode).

Compound	ROOTS					LEAVES				
	Accuracy (%)	Precision (RSD_r_ %)	ME (%)	LOD (ng g^−1^)	LOQ (ng g^−1^)	Accuracy (%)	Precision (RSD_r_ %)	ME (%)	LOD (ng g^−1^)	LOQ (ng g^−1^)
4′-Hydroxydiclofenac	79	6	451	0.32	0.96	108	17	-70	0.09	0.26
4-Nitro-sulfamethoxazole	86	17	109	0.10	0.29	80	4	49	0.10	0.29
5-Desamino 5-oxo-2,5-dihydro Lamotrigine	63	9	-12	0.08	0.25	88	26	-44	0.08	0.25
5-Methyl-2H-benzotriazole	59	8	38	0.07	0.20	147	37	-85	0.07	0.20
Acesulfame	74	2	6	0.12	0.38	60	9	-18	0.12	0.38
Acetaminophen	89	16	421	0.04	0.13	88	6	87	0.04	0.13
Acridone	88	13	-24	0.02	0.05	89	8	-69	0.02	0.05
Benzotriazole	81	15	8	0.06	0.19	80	23	-46	0.04	0.12
Bezafibrate	94	4	27	0.04	0.11	87	7	25	0.03	0.09
Bisphenol A	89	8	9	0.11	0.33	93	11	-29	0.11	0.33
Caffeine	81	6	2	0.21	0.63	81	16	1	0.13	0.40
Carbamazepine	94	7	2	0.03	0.08	72	12	-25	0.02	0.06
Carbamazepine-10,11-epoxide	39	8	54	0.09	0.28	69	3	47	0.07	0.22
Chloramphenicol	96	16	4	0.12	0.36	85	1	47	0.12	0.36
Ciprofloxacin	68	24	-29	0.20	0.61	13	50	-91	0.18	0.53
Citalopram	129	6	-50	0.07	0.21	85	9	-61	0.07	0.21
Clarithromycin	83	8	118	0.01	0.04	76	6	87	0.01	0.04
Climbazole	156	10	-65	0.01	0.02	97	5	-50	0.01	0.02
Clofibric acid	90	6	66	0.03	0.08	92	12	50	0.02	0.06
Diclofenac	79	6	-65	0.09	0.27	119	43	-93	0.11	0.32
Diltiazem	124	4	219	0.15	0.46	84	5	245	0.15	0.46
Fenofibrate	74	16	-93	0.06	0.20	108	25	-99	0.06	0.20
Fluconazole	102	6	3	0.23	0.71	123	4	-32	0.23	0.71
Furosemide	85	16	34	0.07	0.20	112	7	-44	0.09	0.27
Gemfibrozil	77	8	-83	0.15	0.46	98	18	-93	0.15	0.46
Hydrochlorothiazide	86	4	-22	0.01	0.02	95	9	-48	0.05	0.15
Ibuprofen	93	21	-55	0.05	0.15	137	22	-79	0.05	0.15
Indomethacin	91	19	69	0.10	0.31	68	6	-5	0.10	0.31
Irbesartan	122	8	-51	0.04	0.11	67	14	-84	0.06	0.17
Lamotrigine	95	2	-35	0.05	0.14	69	2	-53	0.05	0.14
Lamotrigine N2-oxide	63	15	-27	0.03	0.09	77	7	-58	0.03	0.09
Metoprolol	90	8	36	0.11	0.33	75	7	-14	0.11	0.33
Metronidazole	86	11	27	0.07	0.20	105	6	29	0.10	0.29
N2-Methyl-lamotrigine	104	6	-29	0.03	0.09	74	10	-52	0.03	0.09
N-Acetylsulfamethoxazole	93	6	-20	0.08	0.24	110	6	-21	0.08	0.24
Oxcarbazepine	88	5	205	0.14	0.43	68	11	260	0.10	0.30
Propranolol	133	8	-45	0.05	0.14	100	6	-36	0.05	0.14
Sucralose	87	7	277	0.10	0.30	69	8	156	0.10	0.30
Sulfamethazine	103	16	-64	0.29	0.89	125	7	-72	0.29	0.89
Sulfamethoxazole	98	10	-76	0.31	0.95	127	10	-86	0.24	0.73
Sulfanylamide	80	40	-68	0.04	0.13	106	44	-36	0.04	0.13
Sulfanilic acid	98	7	-75	0.08	0.25	28	29	-85	0.08	0.25
Valsartan	79	17	-18	0.07	0.19	123	88	-94	0.11	0.34
Valsartan acid	97	4	-4	0.11	0.34	92	8	-60	0.11	0.34
Verapamil	140	8	-40	0.17	0.50	99	4	-26	0.17	0.50

There are only few previous studies that used QuEChERS for the extraction of CECs in radish tissues (Table 3) [8,^9^,^26^,27]. To our knowledge there are no studies using HRMS for a such matrix. We only found two studies for detection/identification of metabolites using cell cultures or under hydroponic conditions using a QToF and Ion mobility-QToF, respectively [28,29]. The results of our validated method are comparable to the previous studies in terms of accuracy, ME, LODs and LOQs. All methodologies reported shows the use of the three commercially available salts (Original, European and AOAC) showing great overall recoveries. In all cases, a clean-up step consisting of d-SPE was performed with exclusion of the present study. However, no significant differences were observed in term of ME comparing the ME values reported by Martínez-Piernas et al. ((-87) – 231) and our methods ((-93) – 451) for radish root and ((-99) – 260) for radish leaves. That allows skipping the clean-up step, therefore cheapening the total cost of the method, reducing the sample treatment time and avoiding potential analytes loses due to the use of extra salts. Finally, the acquisition took place using the QToF-MS instrument in MRMHR mode. This acquisition mode shows greater selectivity than LR-MS instruments due to the use of high resolution, and a sensitivity comparable to QQQ and QTrap instruments, resulting in similar LODs and LOQs [30]. The key point here is the development of a specific approach for radish roots and another for radish leaves which improves terms in accuracy, precision and ME.

**Table 3:** Comparison of the study with previous reported QuEChERS methods for determination of CECs in radish crops.

Sample weight (g)	Extraction solvent	Salts	Buffer	Clean-up	N° Analytes	Recovery (%)	LOD (ng g^−1^)	LOQ (ng g^−1^)	ME (%)	MSMS	Ref.
10.0 root and leaves	20 mL ACN 1% Acetic Acid	1 g Na_3_Cit·2H_2_O, 0.5 g Na_2_Cit·5H_2_O, 0.1 g Na_2_-EDTA	-	d-SPE (30 mg PSA, 30 mg C18)	3	79 - 115	0.6-6	2-20	N.R.	QqQ	[9]
0.5 d.w. root and leaves	5 mL ACN / MeOH (65:35, v/v)	2 g Na_2_SO_4_, 1 g NaCl	150 mg Na_2_ EDTA	250 mg Na_2_SO_4_ + d-SPE (25 mg C18, 25 mg PSA)	15	51-104	0.7-6.5	N.R.	N.R.	QqQ	[26]
10.0 root and leaves	10 mL ACN 1% Acetic Acid	6 g of anhydrous MgSO_4_	1.5 g NaOAc	d-SPE (750 mg MgSO_4_, 125 mg C18, 125 mg PSA)	74	25-134	0.01-2	0.02-2	231-(-87)	QTrap	[8]
1.0 d.w. roots and leaves	10 mL ACN	4 g MgSO_4_, 1 g NaCl	-	d-SPE (150 mg PSA, 900 mg MgSO_4_, 45 mg GCB)	3	N.R.	0.3-0.6	N.R.	N.R.	QqQ	[27]
1.0 d.w. root	10 mL ACN	4 g MgSO_4_, 1 g NaCl	-	-	45	39-156	0.01-0.32	0.02-0.96	451-(-83)	QToF	Present study
1.0 d.w. leaves	10 mL ACN 0,1% FA	4 g MgSO_4_, 1 g NaCl	1 g Nacitrate, 0.5 g disodium citrate sesquihydrate	-	45	13-147	0.01-0.29	0.02-0.89	260-(-99)	QToF	

## Method applicability

To test the applicability of the method, radish plants were growth in controlled conditions according to the same procedure reported elsewhere and watered using artificial contaminated water at 10 ng g^−1^. Briefly, Radish seeds were sown in pots (4 seeds per pot, n = 6 pots) and after 5 days from germination, the seedling were regularly irrigated every two days for 20 days with 200 mL of artificial contaminated water at 10 ng g^−1^. Control samples were irrigated only with tap water. The use of fertilizers was needed to ensure a correct crop growth. After 21 days, crops were harvested and hand-washed to remove any soil particles. Then, roots were separated from leaves and individually frozen at -20 °C for 48  h, freeze-dried, and prepared according the validation procedure. Among the validated compounds, ten CECs have been satisfactorily quantified in both radish roots and leaves. Results and frequency of detection (FoD) are reported in Table 4. Four compounds were detected in both, roots and leaves (Climbazole, metoprolol, propranolol, verapamil). While furosemide, gemfibrozil and irbesartan were detected only in the roots, the leaves were merely positive for bisphenol A, ibuprofen and ketoprofen. The compound with the highest accumulation in root was furosemide (6 ng g^−1^), while verapamil in leaves (6 ng g^−1^). Moreover, propranolol (1.1 ng g^−1^) and bisphenol A (1.9 ng g^−1^) are the compounds showing the lowest concentration in roots and leaves, respectively.

**Table 4:** Radish root and leaves concentration results at 10 ng g^−1^ (n = 6).

Compounds	Root (ng g^−1^)	FoD*	Leaves (ng g^−1^)	FoD*
Bisphenol A	n.d.	-	1.9	2
Climbazole	3.2	6	5.3	6
Furosemide	6.0	1	n.d.	-
Gemfibrozil	1.8	3	n.d.	-
Ibuprofen	n.d.	-	2.8	2
Irbesartan	1.2	6	n.d.	-
Ketoprofen	n.d.	-	3.9	1
Metoprolol	3.2	6	5.6	6
Propranolol	1.1	5	3.0	6
Verapamil	4.4	6	6.0	6

*FoD: Frequency of Detection

## Declaration of Competing Interest

The Authors confirm that there are no conflicts of interest.

## Appendix Supplementary materials

Image, application 1
