# Supplementary material for: One-step extraction and analysis of 45 contaminants of emerging concern using QuEChERS methodology and HR-MS in radish leaves and roots
Source: MethodsX. 2021 Mar 15;8:101308. doi: 10.1016/j.mex.2021.101308 (PMC8374258; doi:10.1016/j.mex.2021.101308)
Supplement: Supplementary file 1 [file mmc1.docx]

Supplementary Information

Table S1a.

Relative recoveries (%) and intraday precision (RSD %) at 5 concentrations (2, 5, 10, 50, 200 ng g^-1^) with MRM^HR^ acquisition mode for radish root matrix.

| Compounds | ESI | 2 ng g^-1^  (RSD %) | 5 ng g^-1^  (RSD %) | 10 ng g^-1^  (RSD %) | 50 ng g^-1^  (RSD %) | 200 ng g^-1^  (RSD %) |
| --- | --- | --- | --- | --- | --- | --- |
| Acridone | + | 65 (8.4 %) | 95 (7.6 %) | 88 (12.7 %) | 80 (7.8 %) | 90 (1.4 %) |
| Caffeine | + | 67 (37.8 %) | 101 (13.3 %) | 81 (6.3 %) | 74 (5.5 %) | 79 (2.1 %) |
| Carbamazepine | + | 75 (12.2 %) | 91 (14.3 %) | 94 (7.2 %) | 87 (2.8 %) | 92 (2.0 %) |
| Carbamazepine-10,11-epoxide | + | 66 (5.4 %) | 140 (26.1 %) | 39 (8.3 %) | 55 (31.9 %) | 69 (12.5 %) |
| Ciprofloxacin | + | 35 (26.7 %) | 48 (34.0 %) | 68 (24.4 %) | 47 (13.9 %) | 65 (0.8 %) |
| Citalopram | + | 91 (6.6 %) | 128 (6.0 %) | 129 (6.1 %) | 87 (12.0 %) | 86 (5.6 %) |
| Clarithromycin | + | 67 (7.0 %) | 103 (7.3 %) | 83 (7.7 %) | 72 (7.0 %) | 88 (3.4 %) |
| 5-Desamino 5-Oxo-2,5-dihydro Lamotrigine | + | 77 (60.4 %) | 95 (22.0 %) | 63 (8.8 %) | 82 (10.4 %) | 83 (6.9 %) |
| Diltiazem | + | 80 (17.9 %) | 120 (6.3 %) | 124 (4.0 %) | 93 (1.7 %) | 90 (6.7 %) |
| Fenofibrate | + | 45 (43.8 %) | 94 (39.9 %) | 74 (15.8 %) | 68 (19.9 %) | 64 (23.6 %) |
| Fluconazole | + | 78 (6.3 %) | 111 (6.2 %) | 102 (6.2 %) | 88 (2.2 %) | 89 (4.8 %) |
| Lamotrigine | + | 71 (7.4 %) | 111 (7.3 %) | 95 (1.8 %) | 77 (2.6 %) | 76 (4.1 %) |
| Lamotrigine N2-oxide | + | 32 (66.1 %) | 118 (29.7 %) | 63 (15.4 %) | 170 (7.6 %) | 168 (10.6 %) |
| Metoprolol | + | 55 (15.5 %) | 91 (4.2 %) | 90 (8.1 %) | 74 (7.9 %) | 88 (1.8 %) |
| 5-Methyl-2H-benzotriazole | + | 25 (15.5 %) | 51 (12.6 %) | 59 (8.3 %) | 66 (7.6 %) | 82 (2.4 %) |
| Metronidazole | + | 80 (12.6 %) | 103 (13.0 %) | 86 (11.0 %) | 67 (8.1 %) | 79 (3.5 %) |
| N2-Methyl-Lamotrigine | + | 67 (7.9 %) | 117 (3.8 %) | 104 (6.0 %) | 91 (5.2 %) | 77 (17.5 %) |
| 4-Nitro-sulfamethoxazole | + | 53 (5.0 %) | 91 (1.7 %) | 86 (17.1 %) | 67 (10.7 %) | 94 (1.4 %) |
| Oxcarbazepine | + | 48 (16.2 %) | 104 (7.0 %) | 88 (4.9 %) | 73 (5.6 %) | 75 (20.0 %) |
| Propranolol | + | 72 (29.4 %) | 119 (14.9 %) | 133 (7.8 %) | 84 (14.3 %) | 72 (17.6 %) |
| Sulfamethazine | + | 91 (9.3 %) | 106 (19.4 %) | 103 (15.9 %) | 97 (2.9 %) | 86 (1.6 %) |
| Valsartan acid | + | 80 (32.1 %) | 104 (11.7 %) | 97 (3.7 %) | 80 (9.3 %) | 83 (1.8 %) |
| Verapamil | + | 82 (11.7 %) | 139 (6.9 %) | 140 (7.5 %) | 95 (5.1 %) | 87 (5.9 %) |
| Acesulfame | - | 67 (6.8 %) | 75 (6.2 %) | 75 (2.1 %) | 61 (7.2 %) | 72 (1.9 %) |
| Acetaminophen | - | 69 (49.0 %) | 94 (18.4 %) | 89 (16.2 %) | 79 (15.4 %) | 81 (3.5 %) |
| Benzotriazole | - | 63 (7.2 %) | 102 (27.5 %) | 81 (15.4 %) | 80 (8.2 %) | 83 (4.5 %) |
| Bezafibrate | - | 73 (4.3 %) | 109 (8.1 %) | 94 (3.8 %) | 79 (5.6 %) | 90 (5.7 %) |
| Bisphenol A | - | 47 (17.9 %) | 87 (8.3 %) | 89 (8.1 %) | 76 (1.1 %) | 83 (1.7 %) |
| Chloramphenicol | - | 66 (3.3 %) | 100 (12.4 %) | 96 (15.6 %) | 80 (9.8 %) | 83 (2.5 %) |
| Climbazole | - | 99 (9.7 %) | 186 (13.1 %) | 156 (10.2 %) | 104 (9.5 %) | 73 (21.0 %) |
| Clofibric acid | - | 77 (14.3 %) | 93 (11.8 %) | 90 (5.5 %) | 77 (7.8 %) | 87 (3.4 %) |
| Diclofenac | - | 56 (40.8 %) | 95 (23.3 %) | 79 (5.7 %) | 75 (8.0 %) | 77 (8.7 %) |
| Furosemide | - | 56 (15.1 %) | 73 (1.5 %) | 85 (15.9 %) | 89 (6.5 %) | 106 (1.9 %) |
| Gemfibrozil | - | 67 (34.0 %) | 100 (10.2 %) | 77 (8.4 %) | 79 (5.1 %) | 80 (2.4 %) |
| Hydrochlorothiazide | - | 72 (24.2 %) | 103 (10.9 %) | 87 (4.1 %) | 83 (15.1 %) | 85 (8.7 %) |
| 4'-Hydroxydiclofenac | - | 63 (5.8 %) | 86 (4.8 %) | 79 (6.4 %) | 69 (3.8 %) | 82 (0.9 %) |
| Ibuprofen | - | 69 (41.2 %) | 67 (12.4 %) | 93 (20.6 %) | 72 (12.1 %) | 80 (11.8 %) |
| Indomethacin | - | 70 (20.5 %) | 89 (11.6 %) | 91 (19.0 %) | 76 (9.8 %) | 73 (3.6 %) |
| Irbesartan | - | 75 (14.5 %) | 125 (4.7 %) | 122 (8.4 %) | 78 (1.4 %) | 82 (9.2 %) |
| N-Acetyl-sulfamethoxazole | - | 69 (3.7 %) | 95 (6.8 %) | 93 (5.8 %) | 79 (5.0 %) | 91 (2.2 %) |
| Sucralose | - | 62 (11.5 %) | 89 (2.9 %) | 88 (7.3 %) | 65 (4.1 %) | 78 (3.2 %) |
| Sulfamethoxazole | - | 105 (23.5 %) | 164 (10.1 %) | 98 (10.4 %) | 93 (5.1 %) | 70 (11.8 %) |
| Sulfanilamide | - | 51 (63.2 %) | 113 (34.0 %) | 80 (40.2 %) | 75 (7.1 %) | 75 (2.9 %) |
| Sulfanilic acid | - | 98 (4.1 %) | 89 (10.3 %) | 98 (7.0 %) | 31 (6.7 %) | 15 (25.9 %) |
| Valsartan | - | 82 (12.6 %) | 99 (4.2 %) | 79 (16.9 %) | 67 (6.9 %) | 84 (6.1 %) |

Table S1b.

Relative recoveries and intraday precision (RSD %) at 5 concentrations (2, 5, 10, 50, 200 ng g-1) with MRM^HR^ acquisition mode for radish leaves matrix.

| Compounds | ESI | 2 ng g^-1^  (RSD %) | 5 ng g^-1^  (RSD %) | 10 ng g^-1^  (RSD %) | 50 ng g^-1^  (RSD %) | 200 ng g^-1^  (RSD %) |
| --- | --- | --- | --- | --- | --- | --- |
| Acridone | + | 91 (14.2 %) | 78 (14.4 %) | 89 (7.9 %) | 75 (6.9 %) | 100 (3.0 %) |
| Caffeine | + | 67 (23.6 %) | 66 (10.3 %) | 81 (16.4 %) | 77 (15.2 %) | 82 (3.7 %) |
| Carbamazepine | + | 73 (11.1 %) | 70 (4.1 %) | 72 (12.4 %) | 82 (5.2 %) | 92 (4.5 %) |
| Carbamazepine-10,11-epoxide | + | 78 (6.7 %) | 65 (10.8 %) | 69 (3.4 %) | 68 (5.3 %) | 83 (0.8 %) |
| Ciprofloxacin | + | / | / | 13 (50.5 %) | 14 (42.3 %) | 13 (13.7 %) |
| Citalopram | + | 84 (7.5 %) | 85 (9.6 %) | 85 (9.3 %) | 67 (3.0 %) | 81 (10.1 %) |
| Clarithromycin | + | 77 (8.1 %) | 78 (6.6 %) | 76 (5.8 %) | 69 (1.6 %) | 85 (1.7 %) |
| 5-Desamino 5-Oxo-2,5-dihydro Lamotrigine | + | 57 (26.6 %) | 82 (57.0 %) | 88 (26.3 %) | 72 (11.7 %) | 81 (5.4 %) |
| Diltiazem | + | 74 (7.3 %) | 80 (3.6 %) | 84 (5.1 %) | 77 (4.5 %) | 87 (4.1 %) |
| Fenofibrate | + | 601 (76.4 %) | 80 (46.7 %) | 108 (24.7 %) | 68 (67.0 %) | 146 (64.8 %) |
| Fluconazole | + | 123 (8.1 %) | 129 (11.4 %) | 123 (4.0 %) | 115 (6.8 %) | 105 (7.0 %) |
| Lamotrigine | + | 70 (4.5 %) | 72 (3.3 %) | 69 (1.8 %) | 65 (3.9 %) | 72 (2.2 %) |
| Lamotrigine N2-oxide | + | 50 (35.0 %) | 71 (39.4 %) | 77 (7.1 %) | 136 (18.2 %) | 151 (5.7 %) |
| Metoprolol | + | 89 (6.6 %) | 93 (7.7 %) | 75 (7.1 %) | 87 (14.7 %) | 90 (1.7 %) |
| 5-Methyl-2H-benzotriazole | + | 73 (42.3 %) | 155 (20.3 %) | 147 (37.4 %) | 72 (14.6 %) | 81 (5.6 %) |
| Metronidazole | + | 83 (33.7 %) | 87 (13.2 %) | 105 (6.4 %) | 80 (16.5 %) | 80 (8.4 %) |
| N2-Methyl-Lamotrigine | + | 74 (5.9 %) | 79 (4.2 %) | 74 (10.0 %) | 73 (4.4 %) | 86 (6.6 %) |
| 4-Nitro-sulfamethoxazole | + | 79 (14.0 %) | 87 (5.8 %) | 80 (4.3 %) | 81 (1.6 %) | 98 (3.2 %) |
| Oxcarbazepine | + | 69 (10.2 %) | 82 (15.5 %) | 68 (11.2 %) | 80 (7.4 %) | 81 (1.8 %) |
| Propranolol | + | 88 (13.7 %) | 86 (5.7 %) | 100 (5.6 %) | 70 (6.3 %) | 78 (2.1 %) |
| Sulfamethazine | + | 115 (22.0 %) | 140 (15.9 %) | 125 (7.2 %) | 75 (10.7 %) | 85 (7.5 %) |
| Valsartan acid | + | 109 (18.9 %) | 78 (11.5 %) | 92 (8.1 %) | 77 (12.3 %) | 84 (5.3 %) |
| Verapamil | + | 105 (6.9 %) | 105 (4.9 %) | 99 (3.9 %) | 78 (5.8 %) | 89 (1.9 %) |
| Acesulfame | - | 74 (24.7 %) | 64 (16.1 %) | 60 (9.2 %) | 68 (12.0 %) | 72 (5.9 %) |
| Acetaminophen | - | 71 (14.5 %) | 114 (23.8 %) | 88 (5.6 %) | 84 (11.6 %) | 94 (6.9 %) |
| Benzotriazole | - | 67 (9.4 %) | 89 (6.0 %) | 80 (22.6 %) | 65 (10.7 %) | 85 (10.9 %) |
| Bezafibrate | - | 87 (19.3 %) | 99 (5.3 %) | 88 (6.9 %) | 83 (5.3 %) | 92 (1.7 %) |
| Bisphenol A | - | 85 (17.5 %) | 95 (7.9 %) | 93 (11.1 %) | 80 (7.0 %) | 89 (3.2 %) |
| Chloramphenicol | - | 76 (7.8 %) | 83 (2.5 %) | 85 (0.4 %) | 79 (5.2 %) | 82 (4.2 %) |
| Climbazole | - | 84 (2.9 %) | 82 (16.3 %) | 97 (5.3 %) | 65 (0.1 %) | 77 (5.9 %) |
| Clofibric acid | - | 92 (9.5 %) | 89 (7.1 %) | 92 (12.4 %) | 101 (3.6 %) | 102 (4.3 %) |
| Diclofenac | - | / | / | 119 (42.8 %) | 92 (7.9 %) | 95 (3.0 %) |
| Furosemide | - | 61 (47.6 %) | 90 (10.4 %) | 112 (6.7 %) | 110 (7.2 %) | 112 (6.1 %) |
| Gemfibrozil | - | 111 (33.7 %) | 95 (4.5 %) | 98 (17.7 %) | 84 (6.1 %) | 94 (3.3 %) |
| Hydrochlorothiazide | - | 86 (24.5 %) | 83 (12.0 %) | 95 (9.2 %) | 89 (6.3 %) | 96 (2.7 %) |
| 4'-Hydroxydiclofenac | - | 125 (10.3 %) | 144 (40.2 %) | 108 (16.5 %) | 79 (5.8 %) | 88 (6.4 %) |
| Ibuprofen | - | 67 (29.7 %) | 84 (91.4 %) | 137 (22.3 %) | 86 (19.5 %) | 95 (11.9 %) |
| Indomethacin | - | 98 (13.4 %) | 106 (47.6 %) | 68 (6.3 %) | 66 (4.7 %) | 80 (6.8 %) |
| Irbesartan | - | 47 (28.5 %) | 67 (3.8 %) | 67 (14.1 %) | 33 (15.1 %) | 40 (4.2 %) |
| N-Acetyl-sulfamethoxazole | - | 58 (36.7 %) | 113 (12.3 %) | 110 (6.3 %) | 88 (3.4 %) | 88 (6.2 %) |
| Sucralose | - | 97 (23.8 %) | 98 (9.8 %) | 69 (7.6 %) | 77 (3.4 %) | 87 (1.8 %) |
| Sulfamethoxazole | - | 127 (56.8 %) | 139 (37.8 %) | 127 (10.2 %) | 100 (12.9 %) | 89 (15.2 %) |
| Sulfanilamide | - | 91 (5.9 %) | 97 (27.6 %) | 106 (43.7 %) | 81 (16.5 %) | 116 (9.1 %) |
| Sulfanilic acid | - | 76 (51.8 %) | 43 (36.1 %) | 28 (29.5 %) | 8 (112.9 %) | 3 (4.1 %) |
| Valsartan | - | / | 41 (49.8 %) | 123 (87.8 %) | 46 (25.0 %) | 53 (10.5 %) |

**Table S2a.**

Comparison of Matrix effect (%) for target analytes in MRM^HR^ acquisition mode for radish root matrix.

| **Compounds** | **ESI** | **2 ng g^-1^** | **5 ng g^-1^** | **10 ng g^-1^** | **50 ng g^-1^** | **200 ng g^-1^** |
| --- | --- | --- | --- | --- | --- | --- |
| **Acridone** | + | -4.1 | -14.7 | -24.1 | -16.6 | -15.2 |
| **Caffeine** | + | 31.7 | 3.9 | 2.4 | 0.6 | -9.7 |
| **Carbamazepine** | + | 4.3 | 15.7 | 2.6 | -9.6 | -3.9 |
| **Carbamazepine-10,11-epoxide** | + | -24.8 | -39.1 | 54.2 | -24.5 | -20.4 |
| **Ciprofloxacin** | + | -7.5 | -3.0 | -29.3 | -23.1 | -26.7 |
| **Citalopram** | + | -57.0 | -54.4 | -50.5 | -40.8 | -27.9 |
| **Clarithromycin** | + | 173.5 | 151.7 | 118.4 | 34.8 | 3.3 |
| **5-Desamino 5-Oxo-2,5-dihydro Lamotrigine** | + | -10.4 | -14.4 | -12.3 | -4.7 | -4.2 |
| **Diltiazem** | + | 353.7 | 558.7 | 219.0 | -7.0 | -9.8 |
| **Fenofibrate** | + | -91.2 | -93.7 | -93.4 | -95.1 | -89.8 |
| **Fluconazole** | + | 21.6 | -0.4 | 3.4 | 9.9 | 6.0 |
| **Lamotrigine** | + | -36.5 | -37.9 | -35.3 | -23.3 | -18.4 |
| **Lamotrigine N2-oxide** | + | -20.5 | -34.5 | -27.4 | -39.0 | -25.2 |
| **Metoprolol** | + | 56.7 | 44.6 | 36.5 | 18.6 | 9.9 |
| **5-Methyl-2H-benzotriazole** | + | 158.2 | 74.4 | 38.5 | 0.1 | -13.9 |
| **Metrodinazole** | + | -11.0 | 21.0 | 26.8 | 1.4 | -2.1 |
| **N2-Methyl-Lamotrigine** | + | -0.2 | -32.9 | -28.9 | -24.3 | -29.2 |
| **4-Nitro-sulfamethoxazole** | + | 221.7 | 154.5 | 109.1 | 85.5 | 36.8 |
| **Oxcarbazepine** | + | 77.0 | 387.3 | 205.0 | 57.7 | 17.3 |
| **Propanolol** | + | -15.4 | -42.2 | -44.8 | -35.6 | -31.8 |
| **Sulfamethazine** | + | -70.3 | -63.4 | -64.3 | -68.4 | -67.8 |
| **Valsartan acid** | + | -16.8 | -16.0 | -4.0 | -10.2 | -2.7 |
| **Verapamil** | + | -7.1 | -34.0 | -40.0 | -29.8 | -20.2 |
| **Acesulfame** | - | 14.2 | 31.8 | 5.7 | 13.7 | 8.4 |
| **Acetaminophen** | - | 999.3 | 630.8 | 421.3 | 358.4 | 281.2 |
| **Benzotriazole** | - | 25.9 | 12.3 | 8.1 | 0.9 | -2.0 |
| **Bezafibrate** | - | 18.6 | 26.1 | 27.4 | 19.7 | 0.1 |
| **Bisphenol A** | - | 18.7 | 29.5 | 9.0 | 5.5 | 2.4 |
| **Chloramphenicol** | - | 72.6 | 11.5 | 3.7 | -0.3 | 0.0 |
| **Climbazole** | - | -46.7 | -68.9 | -65.1 | -55.8 | -37.8 |
| **Clorifibric acid** | - | 109.0 | 98.3 | 65.8 | 75.1 | 52.4 |
| **Diclofenac** | - | -70.9 | -66.3 | -65.1 | -61.2 | -60.7 |
| **Furosemide** | - | 67.1 | 52.8 | 33.6 | 25.5 | 22.3 |
| **Gemfibrozil** | - | -75.0 | -81.7 | -82.8 | -81.1 | -69.1 |
| **Hydrochlorothiazide** | - | -10.1 | -29.3 | -22.5 | -18.9 | -6.8 |
| **4'-Hydroxydiclofenac** | - | 176.2 | 408.4 | 451.2 | 696.6 | 596.6 |
| **Ibuprofen** | - | -46.1 | -53.6 | -55.4 | -51.6 | -54.9 |
| **Indomethacine** | - | -3.2 | 258.8 | 69.1 | -56.3 | -67.1 |
| **Irbesartan** | - | -32.5 | -50.9 | -51.5 | -41.6 | -19.5 |
| **N-Acetylsulfamethoxazole** | - | -5.4 | -13.6 | -20.0 | -15.8 | -20.3 |
| **Sucralose** | - | 184.0 | 291.1 | 276.8 | 111.8 | 86.6 |
| **Sulfamethoxazole** | - | -67.7 | -80.4 | -75.8 | -77.3 | -73.3 |
| **Sulfanilamide** | - | -63.8 | -68.9 | -68.1 | -61.3 | -63.2 |
| **Sulfanilic acid** | - | -68.3 | -73.6 | -74.7 | -80.6 | -86.1 |
| **Valsartan** | - | -7.0 | 11.4 | -18.0 | -5.4 | -9.6 |

**Table S2b.**

Comparison of Matrix effect (%) for target analytes in MRM^HR^ acquisition mode for radish leaves matrix.

| **Compounds** | **ESI** | **2 ng g^-1^** | **5 ng g^-1^** | **10 ng g^-1^** | **50 ng g^-1^** | **200 ng g^-1^** |
| --- | --- | --- | --- | --- | --- | --- |
| **Acridone** | + | -64.2 | -55.7 | -69.3 | -64.3 | -43.0 |
| **Caffeine** | + | 23.9 | 32.7 | 0.5 | -7.6 | -6.9 |
| **Carbamazepine** | + | -20.8 | -3.9 | -25.1 | -17.9 | -15.1 |
| **Carbamazepine-10.11-epoxide** | + | -27.3 | 50.9 | 47.0 | -29.6 | -27.8 |
| **Ciprofloxacin** | + | -87.6 | -83.6 | -91.2 | -93.5 | -90.4 |
| **Citalopram** | + | -66.7 | -57.7 | -60.8 | -52.9 | -32.5 |
| **Clarithromycin** | + | 67.2 | 146.7 | 86.9 | 4.9 | -1.6 |
| **5-Desamino 5-Oxo-2.5-dihydro Lamotrigine** | + | -18.6 | -44.2 | -44.8 | -33.4 | -30.4 |
| **Diltiazem** | + | 273.2 | 666.3 | 245.5 | -4.3 | -11.6 |
| **Fenofibrate** | + | -98.6 | -97.8 | -99.0 | -95.9 | -96.6 |
| **Fluconazole** | + | -29.6 | -26.6 | -31.8 | -25.6 | -22.6 |
| **Lamotrigine** | + | -65.6 | -51.5 | -52.7 | -49.2 | -32.1 |
| **Lamotrigine N2-oxide** | + | -84.8 | -56.7 | -57.8 | -62.5 | -53.3 |
| **Metoprolol** | + | -14.6 | -3.7 | -14.0 | -15.8 | -14.6 |
| **5-Methyl-2H-benzotriazole** | + | -70.1 | -75.1 | -84.9 | -70.2 | -66.3 |
| **Metrodinazole** | + | -15.2 | 57.8 | 29.4 | -3.5 | 2.5 |
| **N2-Methyl-Lamotrigine** | + | -53.0 | -48.1 | -52.2 | -51.4 | -54.5 |
| **4-Nitro-sulfamethoxazole** | + | 14.9 | 54.9 | 49.0 | 36.5 | 12.3 |
| **Oxcarbazepine** | + | 23.6 | 367.3 | 260.1 | 44.2 | 15.2 |
| **Propanolol** | + | -24.4 | -22.2 | -36.5 | -25.2 | -33.9 |
| **Sulfamethazine** | + | -64.4 | -67.8 | -72.3 | -57.1 | -52.3 |
| **Valsartan acid** | + | -70.5 | -39.0 | -60.4 | -58.3 | -49.1 |
| **Verapamil** | + | -24.1 | -15.4 | -26.2 | -17.3 | -16.7 |
| **Acesulfame** | - | -53.9 | -21.3 | -18.5 | -26.6 | -24.4 |
| **Acetaminophen** | - | 103.0 | 82.3 | 87.4 | 142.0 | 87.0 |
| **Benzotriazole** | - | -61.4 | -32.3 | -46.0 | -37.7 | -32.5 |
| **Bezafibrate** | - | -8.3 | 33.2 | 25.1 | 12.4 | -6.2 |
| **Bisphenol A** | - | -26.7 | -13.5 | -29.4 | -26.3 | -38.2 |
| **Chloramphenicol** | - | 40.4 | 31.3 | 46.7 | 30.6 | 12.5 |
| **Climbazole** | - | -60.4 | -43.0 | -49.7 | -43.0 | -33.5 |
| **Clorifibric acid** | - | 37.7 | 39.7 | 50.1 | 43.2 | 36.8 |
| **Diclofenac** | - | / | / | -92.6 | -90.1 | -90.8 |
| **Furosemide** | - | -45.6 | -36.1 | -44.5 | -39.0 | -43.7 |
| **Gemfibrozil** | - | -93.3 | -92.2 | -93.3 | -93.4 | -89.8 |
| **Hydrochlorothiazide** | - | -61.7 | -41.7 | -48.3 | -49.0 | -42.3 |
| **4'-Hydroxydiclofenac** | - | -74.0 | -74.6 | -70.5 | -67.1 | -67.8 |
| **Ibuprofen** | - | -75.5 | -65.3 | -78.7 | -72.6 | -75.8 |
| **Indomethacine** | - | -76.8 | 77.5 | -4.8 | -79.8 | -87.2 |
| **Irbesartan** | - | -86.5 | -79.0 | -84.4 | -79.8 | -67.7 |
| **N-Acetylsulfamethoxazole** | - | 0.7 | 1.1 | -21.1 | -10.3 | -16.2 |
| **Sucralose** | - | -22.9 | 149.3 | 155.8 | 48.2 | 31.6 |
| **Sulfamethoxazole** | - | -87.3 | -82.1 | -85.6 | -82.3 | -80.3 |
| **Sulfanilamide** | - | 289.1 | 42.3 | -35.6 | -62.2 | -69.2 |
| **Sulfanilic acid** | - | -93.6 | -90.7 | -85.4 | -83.2 | -76.9 |
| **Valsartan** | - | -88.1 | -82.2 | -93.8 | -82.8 | -81.4 |
